# Evaluation of case definitions to detect respiratory syncytial virus infection in hospitalized children below 5 years in Rural Western Kenya, 2009–2013

**DOI:** 10.1186/s12879-016-1532-0

**Published:** 2016-05-21

**Authors:** Bryan O. Nyawanda, Joshua A. Mott, Henry N. Njuguna, Lilian Mayieka, Sammy Khagayi, Reuben Onkoba, Caroline Makokha, Nancy A. Otieno, Godfrey M. Bigogo, Mark A. Katz, Daniel R. Feikin, Jennifer R. Verani

**Affiliations:** Center for Global Health Research, Kenya Medical Research Institute, Kisumu, Kenya; Division of Global Health Protection, Centers for Disease Control and Prevention, Nairobi, Kenya; Centers for Disease Control and Prevention, Atlanta, GA USA

**Keywords:** Respiratory syncytial virus, Case definitions, Sensitivity and specificity

## Abstract

**Background:**

In order to better understand respiratory syncytial virus (RSV) epidemiology and burden in tropical Africa, optimal case definitions for detection of RSV cases need to be identified.

**Methods:**

We used data collected between September 2009 - August 2013 from children aged <5 years hospitalized with acute respiratory Illness at Siaya County Referral Hospital. We evaluated the sensitivity, specificity, positive predictive value (PPV) and negative predictive value (NPV) of individual signs, symptoms and standard respiratory disease case definitions (severe acute respiratory illness [SARI]; hospitalized influenza-like illness [hILI]; integrated management of childhood illness [IMCI] pneumonia) to detect laboratory-confirmed RSV infection. We also evaluated an alternative case definition of cough or difficulty breathing plus hypoxia, in-drawing, or wheeze.

**Results:**

Among 4714 children hospitalized with ARI, 3810 (81 %) were tested for RSV; and 470 (12 %) were positive. Among individual signs and symptoms, cough alone had the highest sensitivity to detect laboratory-confirmed RSV [96 %, 95 % CI (95–98)]. Hypoxia, wheezing, stridor, nasal flaring and chest wall in-drawing had sensitivities ranging from 8 to 31 %, but had specificities >75 %. Of the standard respiratory case definitions, SARI had the highest sensitivity [83 %, 95 % CI (79–86)] whereas IMCI severe pneumonia had the highest specificity [91 %, 95 % CI (90–92)]. The alternative case definition (cough or difficulty breathing plus hypoxia, in-drawing, or wheeze) had a sensitivity of [55 %, 95 % CI (50–59)] and a specificity of [60 %, 95 % CI (59–62)]. The PPV for all case definitions and individual signs/symptoms ranged from 11 to 20 % while the negative predictive values were >87 %. When we stratified by age <1 year and 1- < 5 years, difficulty breathing, severe pneumonia and the alternative case definition were more sensitive in children aged <1 year [70 % vs. 54 %, *p* < 0.01], [19 % vs. 11 %, *p* = 0.01] and [66 % vs. 43 %, *p* < 0.01] respectively, while non-severe pneumonia was more sensitive [14 % vs. 26 %, *p* < 0.01] among children aged 1- < 5 years.

**Conclusion:**

The sensitivity and specificity of different commonly used case definitions for detecting laboratory-confirmed RSV cases varied widely, while the positive predictive value was consistently low. Optimal choice of case definition will depend upon study context and research objectives.

## Background

Respiratory syncytial virus (RSV) is a leading viral cause of lower respiratory tract infection in infants and young children, and an important cause of neonatal death in Kenya [1–9] and worldwide [10–12]. Testing for RSV is not routinely performed in resource-poor settings, and the utility of clinical case definitions for measuring RSV disease in these contexts is not well described. In Kenya, case definitions designed for other purposes, such as for influenza surveillance, have been used to estimate RSV burden [7, 9]. However, the most sensitive and or specific clinical case definitions for detection of RSV among patients with respiratory illness remain underexplored. In addition, with candidate RSV vaccines in development [2, 13], studies are needed to fully assess RSV disease burden and standardized RSV case definitions will be needed to evaluate vaccine efficacy and impact. An understanding of the performance of different clinical case definitions for identifying RSV infection can guide research and surveillance methodology.

We used data collected between September 2009 - August 2013 from children aged <5 years hospitalized with any respiratory illness at Siaya County Referral Hospital. We evaluated the sensitivity, specificity, positive predictive value (PPV) and negative predictive value (NPV) of individual respiratory signs, symptoms and case definitions used for respiratory disease surveillance, including severe acute respiratory infection (SARI), Integrated Management of Childhood Illness (IMCI) non-severe, severe and very severe pneumonia and a hospitalized influenza like illness (hILI) case definition to detect laboratory-confirmed RSV infections.

## Methods

### Study site and population

The Kenya Medical Research Institute, in partnership with the U.S Centers for Disease Control and Prevention (KEMRI/CDC), has conducted surveillance for hospitalized acute respiratory illness (ARI) at Siaya County Referral Hospital (SCRH) since August 2009. SCRH is an inpatient facility with a bed capacity of 200, located in Karemo Division, Siaya County, in Western Kenya. The population is culturally homogenous and almost entirely rural [14]. The area is endemic for malaria [15] with a high under five mortality ratio (94 deaths per 1000 live births in 2012, KEMRI/CDC unpublished data) and a high HIV prevalence, estimated at 25.3 % among women aged ≥15 years in Siaya County [16]. Antiretroviral treatment is recommended for all HIV-infected children <10 years, and coverage among children in Siaya is estimated to be 43 % [16].

### Data collection and management

Hospitalized acute respiratory infection (ARI) was broadly defined as hospitalization with cough or difficulty breathing or chest pain with an onset within the last 14 days. All children meeting the ARI case definition and whose parents/guardians provided consent were enrolled into the surveillance by trained study staff (nurses and clinical officers). Demographic, clinical, and physical examination data were collected using a structured questionnaire. Axillary temperatures were measured. Data processing and management procedures for this surveillance system have been described previously [17, 18].

### Specimen collection and laboratory

Nasopharyngeal (NP) and oropharyngeal (OP) swabs were collected from enrolled patients using flexible mini-tip flocked swabs, then combined into a single vial containing viral transport media. The samples were transported at 4 °C and once received in the laboratory were stored at −80 °C until testing. The cold chain was maintained throughout the testing procedure. Nucleic acids were extracted from the combined NP/OP swab specimens, using a MagMax viral RNA kit and Kingfisher mL instrument (Life Technologies, New York, NY). The extracted nucleic acids were tested in a 1-step real-time reverse-transcription polymerase chain reaction (RT-PCR) assay with pathogen specific primers and probes, using the AgPath-ID One-step RT-PCR kit (Applied Biosystems, Foster City, CA) for RSV and other viruses. RSV was detected using a fluorescence labelled hydrolysis/Taqman probe at a final concentration of 0.1 μM, and the assay was considered positive for RSV when exponential fluorescence curves crossed the assigned threshold at a threshold cycle value of < 40.0 [9].

### Data analysis

We analysed data on children aged <5 years hospitalized with ARI at SCRH from September 2009 through August 2013. Age specific cut-offs were used to define tachypnea: respiratory rate ≥60 breaths per minute in children <2 months, ≥50 breaths per minute in children 2–11 months, ≥40 breaths per minute for children aged 1- < 5 years [19]. Hypoxia was defined as oxygen saturation <90 % on room air [19].

Standard World Health Organization case definitions were used to classify ARI cases that met the criteria for severe acute respiratory infection (SARI, based on 2013 definition [20]), and very severe, severe and non-severe pneumonia according to the Integrated Management of Childhood Illness (IMCI) guidelines [19]. We also applied an influenza-like illness case definition (hILI, normally used in outpatient settings) to hospitalized patients [20], (Table 1). We further developed an alternative case definition by including all individual signs and symptoms in a logistic regression model. We forced the inclusion of cough and difficulty breathing into the model and used stepwise selection to identify important covariates. The resulting case definition was defined as cough or difficulty breathing plus any of hypoxia, chest in-drawing or wheezing.

**Table 1:** Case definitions

Case definition	All age groups
SARI	An Acute Respiratory Infection with: • History of fever or Measured fever of ≥38 °C • And cough • With an onset within the last 10 days • And requires hospitalization
IMCI non-severe pneumonia^a^	Cough or difficulty breathing with fast breathing. - Fast breathing • <2 months ≥ 60 breaths/minute • 2- > 12 months- ≥ 50breaths/min • 12–59 months- ≥ 40 breaths/min
IMCI severe pneumonia^b^	Cough or difficulty breathing with chest in-drawing when calm
IMCI very severe pneumonia	Cough or difficulty breathing with any one of: - Stridor when calm - Not able to breastfeed/drink - Convulsions - Lethargy - Unconscious - Vomit everything
Hospitalized ILI	A hospitalized Acute Respiratory Infection with: • Measured fever of ≥38 °C • And cough • With an onset within the last 10 days

^a^Excludes those with chest in-drawing and those with danger signs (stridor, unable to drink/breastfeed, convulsions, lethargy, unconsciousness and vomit everything)

^b^Excludes those with danger signs

For each respiratory sign, symptom or case definition, we calculated the risk ratio (RR) and 95 % confidence interval to evaluate their association with laboratory-confirmed RSV, and then evaluated the sensitivity, specificity, PPV and NPV for detection of laboratory-confirmed RSV infections. Case definition performance characteristics were calculated for all children aged <5 years, then stratified into two age groups (Infants aged <1 year and children aged 1- < 5 years). We also stratified our results by RSV peak and low season; RSV peak season was defined as the months with average RSV prevalence above 10 %, these were April, May, June and July. Cumulative sensitivities and specificities of the SARI, non-severe, severe and very severe IMCI pneumonia, and hILI case definitions were calculated for increasing symptom durations (days 1 to 14 after first symptom onset). For the case definitions, symptom duration was defined as the number of days from the earliest onset of cough, difficulty breathing, or fever to specimen collection.

We described demographic characteristics and laboratory outcomes of patients using proportions and means. Proportions were compared across groups using z-test for proportions whereas t-tests were used to compare means of continuous variables. Associations between categorical variables were assessed using chi square test of independence. Tests with *p*-value < 0.05 were considered statistically significant. Analysis was performed using SAS (version 9.2, Cary, NC).

## Results

During September 2009 − August 2013, a total of 4714 children aged <5 years were hospitalized with an ARI. Of these, 3878 (82.3 %) had NP/OP swabs collected; reasons for non-collection (*n* = 836) included refusal (*n* = 464, 55.5 %), death before sample collection (*n* = 156, 18.7 %), transfer to other facilities before sample collection (*n* = 30, 3.6 %), too ill to have sample collected (*n* = 22, 2.6 %), and other (*n* = 164, 19.6 %). Among the 3878 with swabs collected, 3810 (98.3 %) had RSV test results available; the remaining 68 (1.7 %) were not tested in the laboratory because the vials were either open or the transport media volume did not meet the required threshold. RSV was detected in 470 (12.3 %) of the children tested.

The mean age and oxygen saturation was similar between those tested and untested for RSV, (mean age 1.4 years vs. 1.4 years, *p* = 0.99) and (mean oxygen saturation 93.7 vs. 93.4, *p* = 0.26) respectively. Of those tested for RSV, 54.0 % were males compared to 58.0 % of the untested (*p* = 0.03). Approximately 3 % (103/3810) of those tested for RSV died compared to 19.6 % (177/904) of ARI cases not tested for RSV (*p* < 0.01), (Table 2).

**Table 2:** Demographic and clinical characteristics of patients tested and not tested for RSV

	Tested *N* = 3810	Untested *N* = 904	*p*-value
	n (%)	n (%)	
Patient characteristics			
Age	(*n* = 3810)	(*n* = 904)	
< 1 years	1756(46.1)	418(46.2)	0.94
1- < 5 years	2054(53.9)	486(53.8)	
Mean age (years)^a^	1.4(1.1)	1.4(1.1)	0.99
Gender	(*n* = 3810)	(*n* = 904)	
Male	2056(54.0)	524(58.0)	0.03
Illness Severity			
Death	(*n* = 3810)	(*n* = 904)	
Discharged dead	103(2.7)	177(19.6)	<0.01
Oxygen saturation	(*n* = 3796)	(*n* = 900)	
< 90 %	628(16.5)	157(17.4)	0.52
Mean Oxygen saturation^a^	93.7(6.8)	93.4(7.6)	0.26

^a^Mean(sd) for the continuous variables

Slightly more than half 238 (50.6 %) of RSV-positive cases were aged <1 year, (Table 3) and more than three-quarters of all cases were aged <2 years, 360 (76.6 %). Cough, difficulty breathing, night sweats, hypoxia, wheezing, stridor, nasal flaring and chest wall in-drawing were significantly associated with having a laboratory-confirmed RSV infection, (Table 3). The symptom/sign most strongly associated with RSV was cough (RR, 2.56; 95 % CI, 1.60-4.10), (Table 3). The presence of fever (measured or reported) was not associated with RSV infection.

**Table 3:** Demographic characteristics, signs, symptoms and case definitions associated with laboratory confirmed RSV infections

	RSV + ve		RSV -ve		Crude RR	*p*-value
	*n* = 470		*n* = 3340		(95 % CI)	
Demographic characteristics	n	%	n	%		
Age group						
< 1 year	238	50.6	1518	45.4	Ref	
1- < 5 years	232	49.4	1822	54.6	0.83(0.70,0.99)	0.03
Sex						
Male	254	54.0	1802	54.0	Ref	
Female	216	46.0	1538	46.0	1.00(0.84,1.18)	0.97
HIV status						
Positive	24	5.1	152	4.6	Ref	
Negative	240	51.1	1728	51.7	0.89(0.61,1.32)	0.57
Unknown	206	43.8	1460	43.7	0.91(0.61,1.34)	0.63
Sign, symptoms and case definitions						
Any fever	424	90.2	2984	89.3	1.09(0.82,1.45)	0.56
Measured fever ≥38 °C	126	26.8	797	23.9	1.15(0.95,1.39)	0.16
Reported fever	418	88.9	2957	88.5	1.04(0.79,1.36)	0.8
Cough	453	96.4	3023	90.5	2.56(1.60,4.10)	<0.01
Difficulty breathing	291	61.9	1715	51.3	1.46(1.23,1.74)	<0.01
Chest pain	1	0.2	23	0.7	0.34(0.05,2.30)	0.27
Night sweats	337	71.7	2224	66.6	1.24(1.02,1.49)	0.03
Tachypnea^a^	288	61.3	1948	58.3	1.11(0.94,1.33)	0.22
Hypoxia^b^	121	26.2	507	15.6	1.76(1.46,2.12)	<0.01
Wheeze	125	26.6	569	17.0	1.63(1.35,1.96)	<0.01
Stridor	36	7.7	142	4.3	1.69(1.25,2.30)	<0.01
Nasal flaring	146	31.1	739	22.1	1.49(1.24,1.78)	<0.01
Chest In-drawing	145	30.9	699	20.9	1.57(1.31,1.88)	<0.01
SARI	389	82.8	2579	77.2	1.36(1.09,1.71)	0.01
ILI	112	23.8	678	20.3	1.20(0.98,1.46)	0.08
IMCI Non-severe pneumonia	95	20.2	777	23.3	0.85(0.69,1.06)	0.14
IMCI severe pneumonia	71	15.1	310	9.3	1.60(1.27,2.01)	<0.01
IMCI very severe pneumonia	236	50.2	1719	51.5	0.96(0.81,1.13)	0.61
Cough or difficulty breathing plus hypoxia, chest in-drawing or wheeze	257	54.7	1316	39.4	1.72(1.15,2.03)	<0.01

^a^Defined as respiratory rate ≥60 breaths per minute in kids <2 months, ≥50 breaths per minute in kids 2–11 months, ≥40 breaths per minute for kids 1- < 5 years

^b^Defined as oxygen saturation <90 % on room air. 14/3810 missing oxygen saturation

Among the respiratory signs and symptoms examined, cough alone had the highest sensitivity for laboratory confirmed RSV (96.4 %, 95 % CI 94.7-98.1), followed by night sweats, difficulty breathing and tachypnea; (71.7 %, 95 % CI 67.6-75.8), (61.9 %, 95 % CI 57.5-66.3) and (61.3 %, 95 % CI 56.9-65.7) respectively. The other individual signs and symptoms evaluated had sensitivities that ranged from 8–31 %, (Table 4). The presence of hypoxia, wheezing, stridor, nasal flaring and chest wall in-drawing all had specificities >77 % for laboratory confirmed RSV (Table 4).

**Table 4:** Sensitivity, specificity, PPV and NPV of signs, symptoms and case definitions

Age group	Case definition	RSV positive *n* = 470		RSV negative *n* = 3340		Sensitivity %(95 % CI)	Specificity %(95 % CI)	PPV %(95 % CI)	NPV %(95 % CI)
All under 5		n	%	n	%				
	Cough	453	96.4	3023	90.5	96.4(94.7,98.1)	9.5(8.5,10.5)	13.0(11.9,14.2)	94.9(92.6,97.3)
	Difficulty breathing	291	61.9	1715	51.3	61.9(57.5,66.3)	48.7(47.0,50.4)	14.5(13.0,16.1)	90.1(88.7,91.5)
	Night sweats	337	71.7	2224	66.6	71.7(67.6,75.8)	33.4(31.8,35.0)	13.2(11.9,14.5)	89.4(87.6,91.1)
	Tachypnea	288	61.3	1948	58.3	61.3(56.9,65.7)	41.7(40.0,43.4)	12.9(11.5,14.3)	88.4(86.9,90.0)
	Hypoxia	121	25.9	507	15.2	25.9(21.9,29.8)	84.8(83.5,86.0)	19.3(16.2,22.4)	89.1(88.0,90.1)
	Wheeze	125	26.6	569	17.0	26.6(22.6,30.6)	83.0(81.7,84.2)	18.0(15.2,20.9)	88.9(87.8,90.0)
	Stridor	36	7.7	142	4.3	7.7(5.3,10.1)	95.8(95.1,96.4)	20.2(14.3,26.1)	88.1(87.0,89.1)
	Nasal flaring	146	31.1	739	22.1	31.1(26.9,35.3)	77.9(76.5,79.3)	16.5(14.1,18.9)	88.9(87.8,90.1)
	Chest In-drawing	145	30.9	699	20.9	30.9(26.7,35.0)	79.1(77.7,80.5)	17.2(14.6,19.7)	89.0(87.9,90.2)
	SARI	389	82.8	2579	77.2	82.8(79.4,86.2)	22.8(21.4,24.2)	13.1(11.9,14.3)	90.4(88.4,92.4)
	hILI	112	23.8	678	20.3	23.8(20.0,27.7)	79.7(78.3,81.1)	14.2(11.7,16.6)	88.2(87.0,89.3)
	IMCI Non-severe pneumonia	95	20.2	777	23.3	20.2(16.6,23.8)	76.7(75.3,78.2)	10.9(8.8,13.0)	87.2(86.0,88.4)
	IMCI severe pneumonia	71	15.1	310	9.3	15.1(11.9,18.3)	90.7(89.7,91.7)	18.6(14.7,22.6)	88.4(87.3,89.4)
	IMCI very severe pneumonia	236	50.2	1719	51.5	50.2(45.7,54.7)	48.5(46.8,50.2)	12.1(10.6,13.5)	87.4(85.9,88.9)
	Cough or difficulty breathing plus hypoxia, chest in-drawing or wheeze	257	54.7	1316	39.4	54.7(50.2,59.2)	60.1(58.9,62.3)	16.3(14.5,18.2)	90.5(89.3,91.7)
Children < 1 years	n	238		1518					
	Cough	232	97.5	1373	90.4	97.5(95.5,99.5)	9.6(8.1,11.0)	14.5(12.7,16.2)	96.0(92.9,99.1)
	Difficulty breathing	166	69.8	863	56.8	69.8(63.9,75.6)^a^	43.2(40.7,45.6)^a^	16.1(13.9,18.4)	90.1(87.9,92.3)
	SARI	189	79.4	1175	77.4	79.4(74.3,84.6)	22.6(20.5,24.7)	13.9(12.0,15.7)	87.5(84.2,90.8)
	hILI	52	21.9	307	20.2	21.9(16.6,27.1)	79.8(77.8,81.8)	14.5(10.8,18.1)	86.7(84.9,89.5)
	IMCI Non-severe pneumonia	34	14.3	312	20.6	14.3(9.8,18.7)^a^	79.5(77.4,81.5)^a^	9.8(6.7,13.0)	85.5(83.7,87.4)
	IMCI severe pneumonia	46	19.3	175	11.5	19.3(14.3,24.3)^a^	88.5(86.9,90.1)^a^	20.8(15.5,26.2)	87.5(85.8,89.2)
	IMCI very severe pneumonia	117	49.2	726	47.8	49.2(42.8,55.5)	52.2(49.7,54.7)	13.9(11.6,16.2)	86.8(84.6,89.0)
	Cough or difficulty breathing plus hypoxia, chest in-drawing or wheeze	157	66.0	698	46.0	66.0(60.0,72.0)^a^	54.0(51.5,56.5)^a^	18.4(15.8,21.0)	91.0(89.1,92.9)
Children 1- < 5 years	n	232		1822					
	Cough	221	95.3	1650	90.6	95.3(92.5,98.0)	9.4(8.1,10.8)	11.8(10.4,13.3)	94.0(90.6,97.4)
	Difficulty breathing	125	53.9	852	46.8	53.9(47.5,60.3)^a^	53.2(51.0,55.5)^a^	12.8(10.7,14.9)	90.0(88.3,91.9)
	SARI	200	86.2	1404	77.1	86.2(81.8,90.6)	22.9(21.0,24.9)	12.5(10.9,14.1)	92.9(90.5,95.3)
	hILI	60	25.9	371	20.4	25.9(20.2,31.5)	79.6(77.8,81.5)	13.9(10.7,17.2)	89.4(87.9,90.9)
	IMCI Non-severe pneumonia	61	26.3	465	25.5	26.3(20.6,32.0)^a^	74.5(72.5,76.5)^a^	11.6(8.9,14.3)	88.8(87.2,90.4)
	IMCI severe pneumonia	25	10.8	135	7.4	10.8(6.8,14.8)^a^	92.6(91.4,93.8)^a^	15.6(10.0,21.3)	89.1(87.7,90.5)
	IMCI very severe pneumonia	119	51.3	993	54.5	51.3(44.9,57.7)	45.5(43.2,47.8)	10.7(8.9,12.5)	88.0(85.9,90.1)
	Cough or difficulty breathing plus hypoxia, chest in-drawing or wheeze	100	43.1	618	33.9	43.1(36.7,495)^a^	66.1(63.9,68.3)^a^	13.9(11.4,16.5)	90.1(88.5,91.7)

^a^Sensitivity and specificity significantly different between the two age groups

**Fig. 1:**
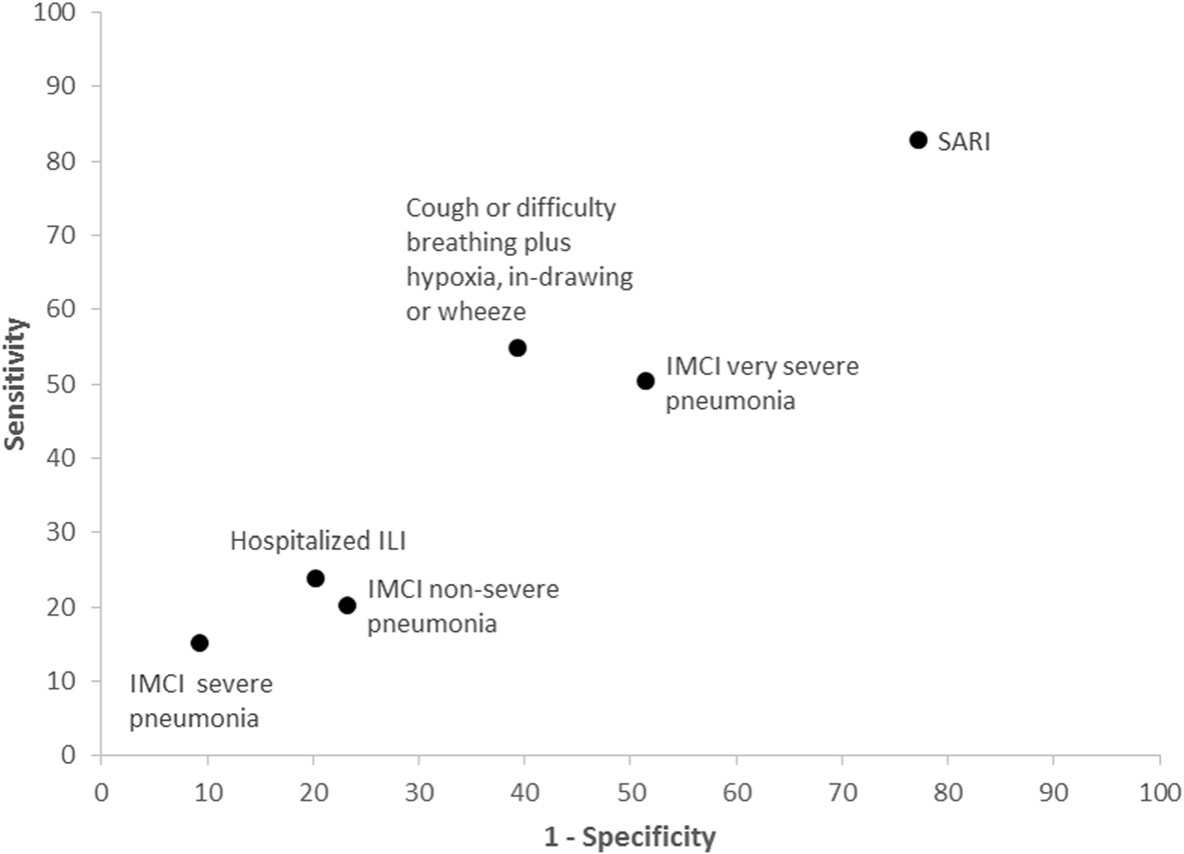
Receiver operator characteristic (ROC) chart for case definitions

The standard respiratory case definition with the highest sensitivity for detecting RSV infection was SARI (82.8 %, 95 % CI 79.4-86.2), followed by IMCI very severe pneumonia (50.2 %, 95 % CI 45.7-54.7), (Table 4. Fig. 1). The sensitivities for IMCI non-severe pneumonia, IMCI severe pneumonia and hILI were lower; (20.2 %, 95 % CI 16.6-23.8), (15.1 %, 95 % CI 11.9-18.3) and (23.8 %, 95 % CI 20.0-27.7) respectively. The highest specificity was observed in IMCI severe pneumonia (90.7 %, 95 % CI 89.7-91.7), closely followed by hILI and IMCI non-severe pneumonia definitions; (79.7 %, 95 % CI 78.3-81.1) and (76.7 %, 95 % CI 75.3-78.2) respectively. SARI and IMCI very severe pneumonia had specificities of 22.8 % (95 % CI 21.4-24.2) and 48.5 % (95 % CI 46.8-50.2), respectively. The alternative case definition developed through logistic regression; cough or difficulty breathing plus hypoxia, chest in-drawing, or wheeze had a sensitivity and specificity of (54.7 %, 95 % CI 50.2-59.2) and (60.1 %, 95 % CI 58.9-62.3) respectively. We plotted a receiver operator characteristics chart for these case definitions, (Fig. 1). The PPV for all case definitions, signs and symptoms assessed ranged between 11–20 % whereas the negative predictive values were >87 %, (Table 4).

**Fig. 2:**
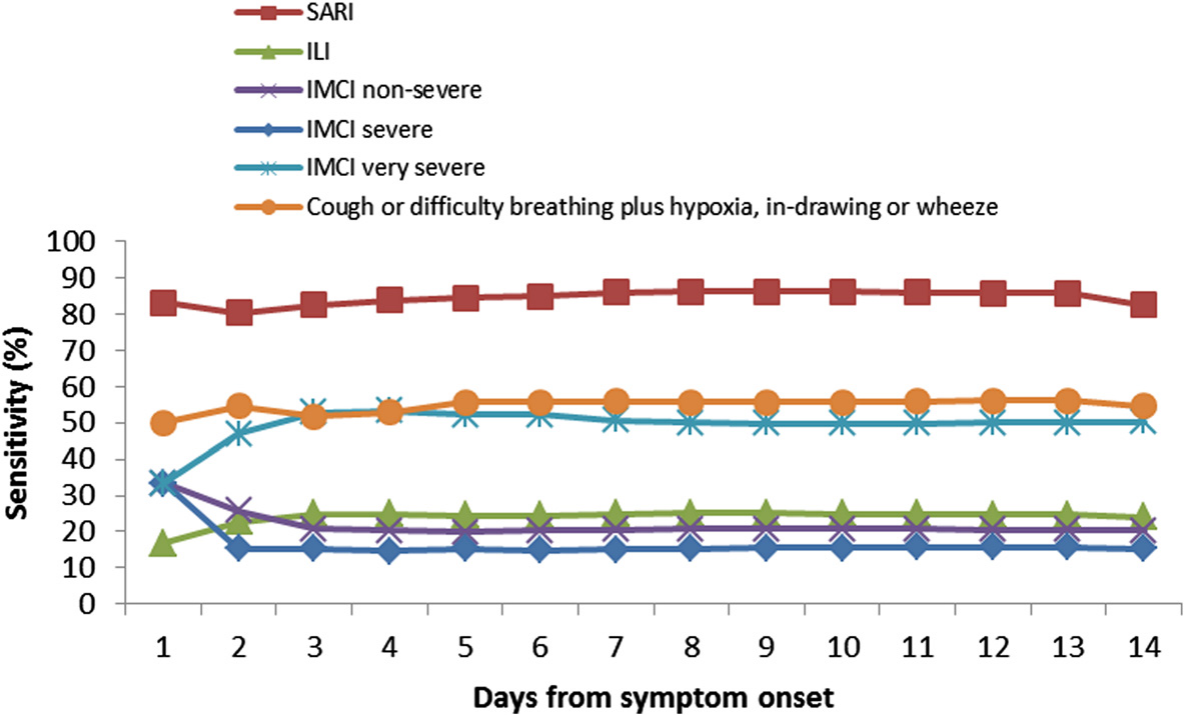
Cumulative sensitivity of case definitions to detect Laboratory confirmed RSV

**Fig. 3:**
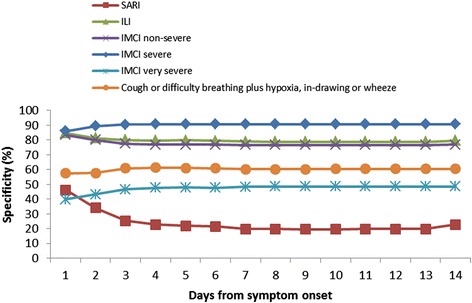
Cumulative specificity of case definitions to detect Laboratory confirmed RSV

When stratified by age group, SARI continued to be the most sensitive case definition (79.4 % sensitive in infants aged <1 year and 86.2 % sensitive in children aged 1- < 5 years), and IMCI severe pneumonia was the most specific (88.5 % in infants aged <1 year and 92.6 % in children aged 1- < 5 years). Comparing across age groups, IMCI severe pneumonia was significantly more sensitive (19.3 % vs. 10.8 %, *p* = 0.01) and less specific (88.5 % vs. 92.6 %, *p* < 0.01) among those aged <1 year compared to children aged 1- < 5 years (Table 4). Cough or difficulty breathing plus hypoxia, chest in-drawing or wheeze was also significantly more sensitive (66.0 % vs. 43.1 %, *p* < 0.01) and less specific (54.0 % vs. 66.1 %, *p* < 0.01) among the younger age group. Difficulty breathing was also significantly more sensitive (69.8 % vs. 53.9 %, *p* < 0.01) and less specific (43.2 % vs. 53.2 %, *p* < 0.01) among the younger age group. IMCI non-severe pneumonia was significantly less sensitive (14.3 % vs. 26.3 %, *p* < 0.01) and more specific (79.5 % vs. 74.5 %, *p* < 0.01) among infants aged <1 year compared to children aged 1- < 5 years, (Table 4). There was no significant difference in sensitivity, specificity and predictive values for the other case definitions between the <1 and 1- < 5 age groups. When we stratified by season, we observed significant difference in the PPVs for all signs, symptoms and case definitions evaluated, with significantly higher PPVs in the RSV peak season, (Table 5). There was no statistically significant difference in the sensitivities and specificities of the case definitions by number of days from symptom onset, (Figs. 2 and 3) except for SARI where specificity decreased from day 1 to 3 (46.2–25.4 %, *p* < 0.01) then stabilized, (Fig. 3).

**Table 5:** PPVs and NPVs of signs, symptoms and case definitions by season

RSV seasons	Case definition	PPV %(95 % CI)	NPV %(95 % CI)
Peak seasons			
	Cough	22.6(20.3,24.9)	91.4(86.1,96.8)
	Difficulty in breathing	26.3(23.1,29.6)	83.8(81.0,86.7)
	SARI	22.8(20.3,25.3)	83.2(78.9,87.5)
	hILI	26.5(21.1,31.9)	79.7(77.3,82.1)
	IMCI Non-severe pneumonia	19.8(15.1,24.5)	78.1(75.6,80.5)
	IMCI severe pneumonia	35.4(27.6,43.2)	80.2(77.9,82.4)
	IMCI very severe pneumonia	19.9(16.9,22.8)	76.6(73.3,79.9)
	Cough or difficulty breathing plus hypoxia, chest in-drawing or wheeze	28.3(24.6,31.9)	83.6(81.0,86.2)
Low seasons			
	Cough	7.7(6.6,8.8)	96.5(94.1,98.9)
	Difficulty in breathing	8.1(6.6,9.6)	93.5(92.1,94.9)
	SARI	7.8(6.6,9.0)	94.2(92.2,96.2)
	hILI	8.3(5.9,10.6)	92.9(91.8,94.1)
	IMCI Non-severe pneumonia	6.8(4.8,8.9)	92.5(91.3,93.7)
	IMCI severe pneumonia	8.4(4.9,12.0)	92.8(91.7,93.9)
	IMCI very severe pneumonia	7.6(6.1,9.1)	92.9(91.5,94.3)
	Cough or difficulty breathing plus hypoxia, chest in-drawing or wheeze	9.4(7.6,11.3)	94.1(92.9,95.3)

## Discussion

We observed wide variations in the sensitivity and specificity of the signs, symptoms and case definitions evaluated. SARI, a case definition requiring cough and either reported or measured fever and developed for global influenza surveillance was the most sensitive case definition for RSV detection with sensitivity of 83 %. The most specific case definition was IMCI severe pneumonia (cough or difficulty breathing plus chest in-drawing). Generally there was a trade-off between sensitivity and specificity. Cough or difficulty breathing plus hypoxia, chest in-drawing or wheeze provided the best balance; it was the only case definition for which both sensitivity and specificity exceeded 50 %. The predictive values were more consistent across case definitions, with all having relatively low PPV (11–20 %) and high NPV (87–90 %).

In determining the optimal case definition, consideration should be given to the context in which the case definition applies and the objectives for which it is being used. If the primary objective of the study is to estimate the burden of RSV disease then it is important to use a case definition offering high sensitivity. We found that cough alone had the highest sensitivity (96 %) to detect RSV. If the goal was to detect the maximum number of RSV cases then cough alone might be the best definition to use. However, using cough alone would also give very many false positives (PPV 13 %). Of the case definitions evaluated we found that the 2013 WHO SARI case definition was most sensitive for RSV detection. Nonetheless, the SARI case definition may miss up to 17 % of RSV cases. Much of what is known about RSV globally comes from surveillance systems that utilize case definitions intended for other diseases (e.g. influenza, pneumonia) that are not optimized for detection of RSV, the potential for underestimation should therefore be recognized in interpreting data on RSV burden, particularly if using a case definition that requires measured fever (e.g. hILI) or includes only pneumonia without danger signs (e.g. IMCI non-severe or severe pneumonia).

Sometimes maximum specificity is desired for RSV detection. If RSV vaccines currently under development are eventually introduced into routine immunization programs there will be a need to evaluate the impact and effectiveness of the vaccine. Given that RSV testing is not widely available in resource poor settings, a clinical case definition may be most practical for use in vaccine evaluations such as case–control vaccine effectiveness studies or analyses of trends in disease burden. For this purpose, a highly specific case definition is preferable [21, 22]. We found that the presence of stridor offered the highest specificity (96 %). However, it was present in < 8 % of cases, and therefore not easily embraceable as a case definition. Among the case definitions evaluated, we found that IMCI severe pneumonia was the most specific (91 %), while hILI and non-severe pneumonia case definitions also provided reasonable specificity (77–80 %). Measuring vaccine impact and effectiveness using the IMCI severe pneumonia case definitions may be more feasible than using less specific case definitions such as SARI. However, even if the most specific case definition were used, most cases identified may not be RSV-infected because all the case definitions were found to have poor PPV.

We observed positive predictive values (PPVs) ranging from 11–20 % for all signs, symptoms and case definitions, though the PPVs were significantly higher during periods of RSV circulation (cold months of the year in Western Kenya; April through July). A case definition with a high PPV is useful to investigators aiming to maximize the detection of laboratory confirmed cases among those tested. For example, studies focused on subtyping or sequencing for which the positive RSV isolate is essential may benefit from a case definition with optimal PPV. In this analysis, the case definition with the highest PPV for detection of laboratory confirmed RSV was IMCI severe pneumonia. However, even during peak RSV season, roughly one in three children with IMCI severe pneumonia will have a positive RSV test result.

Although the prevalence of RSV infection is generally higher among infants hospitalized with respiratory disease compared with older children hospitalized with respiratory disease [1], we did not observe significant differences in PPV when stratifying by age group. Difficulty breathing, severe pneumonia and the alternative definition were found to be more sensitive among the infants aged <1 year, while IMCI non-severe pneumonia was more sensitive among those aged 1- < 5 years. Older children with RSV are likely to be experiencing a reinfection rather than a primary infection, and therefore may present with milder illness [4]. These findings highlight that choice of case definition for detecting RSV cases should take into account the age group under surveillance.

We found that sensitivity, specificity and predictive values for the case definitions to detect RSV infections did not vary significantly with respect to duration from illness onset to sample collection, suggesting that these case definitions can be used within the 14-day period from symptom onset to detect RSV infection without concern about loss of sensitivity or specificity.

The presence of wheezing, hypoxia, stridor, nasal flaring and chest wall in-drawing were each found to be significantly associated with RSV infection. This finding is similar to findings from Rarieda and Kilifi districts in Kenya and Ballabgarh in northern India [1, 6, 9, 23] and suggest that some of the individual IMCI danger signs might be used to identify subsets of children more likely to have RSV virus infections. It should however be noted that there is some variation in the literature regarding the association of hypoxia with RSV infections. Hypoxia was found to be negatively associated with RSV infection in one study conducted in Kilifi District, Kenya [1], with another study reporting no significant association from the same site [6]. We found that neither measured nor reported fever were associated with RSV infection, however history of fever was a common symptom observed in nearly 90 % of children infected with RSV. Fever was a common symptom in many RSV infected patients in other studies as well [23–25] with one study [6] observing that fever was significantly more common among the RSV-infected children compared to children with respiratory disease that tested negative for RSV, RR = 1.54(95 % CI, 1.33–1.80). The high prevalence of malaria in our study site [14, 15] may impact the observed association between fever and RSV.

Interestingly, the performance of various case definitions for detecting RSV infection that we observed in this study differed substantially from the findings of a study in Ballabgarh, northern India, for which all hospitalized children aged <5 years were tested for RSV [23]. While we found the SARI case definition to be the most sensitive, and severe IMCI pneumonia to be most specific, that study reported SARI to have a low sensitivity and IMCI pneumonia or severe pneumonia (combined) to have a low specificity. Differences in epidemiologic contexts (e.g. malaria or HIV prevalence) may explain some of the variation in results. The precise definitions used for SARI and IMCI pneumonia also differed across the two studies. Furthermore, the study from India included all hospitalized children (regardless of clinical signs), while our study included only children hospitalized with cough or difficulty breathing or chest pain. Of note, the Indian study found that restricting to even a very broad respiratory case definition (acute onset cough or sore throat or shortness of breath or coryza or clinician judgement that illness due to infection) would fail to detect 13.5 % (10/74) of RSV cases; thus our analysis likely failed to include some children hospitalized with RSV infection.

In interpreting our results, the following additional limitations should be taken into consideration. This analysis was restricted to inpatients; the findings therefore may not be relevant for outpatient respiratory disease surveillance. Furthermore, because non-severe IMCI pneumonia is typically managed in the outpatient setting in Kenya, those children with non-severe pneumonia included in this analysis may be a somewhat biased group. In addition, a substantial number of severe cases were not swabbed and consequently excluded from this analysis; therefore these findings may not be representative of the most severe spectrum of cases with RSV infection. Lastly, RT-PCR was the only method used for RSV detection in our surveillance system; therefore sensitivities and specificities could vary in studies where other methods such as culture or serology are used.

## Conclusion

The optimal case definition for use in detecting RSV varies depending upon context. Researchers must take into account their objectives, available resources, and the study population. For estimating the burden of RSV in a context similar to Western Kenya, the WHO SARI case definition could be used, particularly if it is already being implemented in a sentinel influenza surveillance system. However more specific case definitions may be required for evaluating future RSV vaccines in settings where diagnostic testing is not readily available. Additional research to assess the performance of various case definitions for detecting RSV infections across a broad range of epidemiologic contexts is needed.

### Ethical considerations

This study was approved by the Ethical Review Committee of the Kenya Medical Research Institute (KEMRI SSC #1801) and the Institutional Review Board of CDC-Atlanta (CDC IRB #3308). Written informed consent was obtained from all caretakers/guardians of all minors prior to enrolment in the surveillance system. All patients admitted at the SCRH were given standard care treatment irrespective of their enrolment into the study or not.

## Abbreviations

ARI: acute respiratory illness
CDC: U.S Centers for Disease Control and Prevention
hILI: hospitalized influenza like illness
HIV: human immunodeficiency virus
IMCI: integrated management of childhood illness
KEMRI: Kenya Medical Research Institute
NPV: negative predictive value
PPV: positive predictive value
RR: relative risk
RSV: respiratory syncytial virus
RT-PCR: real-time reverse-transcription polymerase chain reaction
SARI: severe acute respiratory infection
SAS: statistical analysis software
SCRH: Siaya County Referral Hospital
WHO: World Health Organisation
